# Bis(μ-2-{[oxido(phenyl)methylidene]hydrazinylidene}propanoato)bis[dibenzyl(ethanol)tin(IV)]

**DOI:** 10.1107/S1600536811011937

**Published:** 2011-04-07

**Authors:** Shaojun Sun, Jie Yang

**Affiliations:** a Clinical Laboratory, Liaocheng Hospital, Liaocheng 252000, People’s Republic of China; b Chinese Medicine Hospital of Liaocheng, Liaocheng 252000, People’s Republic of China

## Abstract

In the title complex, [Sn_2_(C_7_H_7_)_4_(C_10_H_8_N_2_O_3_)_2_(C_2_H_5_OH)_2_], the Sn(IV) atom is seven-coordinated in a distorted penta­gonal–bipyramidal geometry by three O atoms and one N atom from the pyruvate benzoyl hydrazone ligand, one ethanol O atom and two axial C atoms from *trans-*benzyl groups, thus forming a dimeric mol­ecule (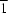 symmetry) *via* weak Sn—O inter­actions. The C atoms of one phenyl ring and the ethanol mol­ecule are disordered over two sets of sites with site-occupancy factors of 0.57 (5):0.43 (5) and 0.79 (2):0.21 (2), respectively. Intermolecular O—H⋯O hydrogen bonds stabilize the crystal structure.

## Related literature

For related structures, see: Sun & Hu (2007 ▶); Gielen *et al.* (2002 ▶).
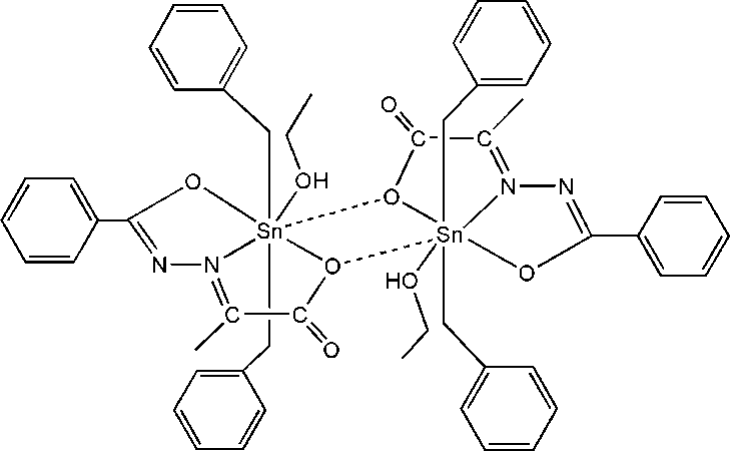


## Experimental

### 

#### Crystal data

[Sn_2_(C_7_H_7_)_4_(C_10_H_8_N_2_O_3_)_2_(C_2_H_6_O)_2_]
*M* *_r_* = 1102.42
Triclinic, 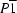

*a* = 8.7187 (18) Å
*b* = 11.385 (2) Å
*c* = 13.198 (3) Å
α = 96.170 (3)°
β = 93.728 (2)°
γ = 105.861 (3)°
*V* = 1246.8 (4) Å^3^
*Z* = 1
Mo *K*α radiation
μ = 1.06 mm^−1^
*T* = 298 K
0.45 × 0.37 × 0.23 mm

#### Data collection

Siemens SMART CCD area-detector diffractometer
Absorption correction: multi-scan (*SADABS*; Sheldrick, 1996 ▶) *T*_min_ = 0.647, *T*_max_ = 0.793
6566 measured reflections
4356 independent reflections
3598 reflections with *I* > 2σ(*I*)
*R*_int_ = 0.017

#### Refinement

*R*[*F* ^2^ > 2σ(*F* ^2^)] = 0.042
*wR*(*F* ^2^) = 0.116
*S* = 1.08
4356 reflections
298 parameters
H-atom parameters constrained
Δρ_max_ = 1.04 e Å^−3^
Δρ_min_ = −0.58 e Å^−3^

### 

Data collection: *SMART* (Siemens, 1996 ▶); cell refinement: *SAINT* (Siemens, 1996 ▶); data reduction: *SAINT*; program(s) used to solve structure: *SHELXS97* (Sheldrick, 2008 ▶); program(s) used to refine structure: *SHELXL97* (Sheldrick, 2008 ▶); molecular graphics: *SHELXTL* (Sheldrick, 2008 ▶); software used to prepare material for publication: *SHELXTL*.

## Supplementary Material

Crystal structure: contains datablocks I, global. DOI: 10.1107/S1600536811011937/lr2004sup1.cif

Structure factors: contains datablocks I. DOI: 10.1107/S1600536811011937/lr2004Isup2.hkl

Additional supplementary materials:  crystallographic information; 3D view; checkCIF report

## Figures and Tables

**Table:** 

Sn1—C11	2.135 (6)
Sn1—O3	2.148 (3)
Sn1—C18	2.154 (6)
Sn1—N1	2.237 (4)
Sn1—O1	2.341 (3)
Sn1—O4	2.382 (4)
Sn1—O1^i^	2.772 (3)

**Table:** 

C11—Sn1—O3	97.42 (19)
C11—Sn1—C18	163.3 (2)
O3—Sn1—C18	94.77 (18)
C11—Sn1—N1	97.9 (2)
O3—Sn1—N1	70.83 (13)
C18—Sn1—N1	96.85 (18)
C11—Sn1—O1	88.59 (19)
O3—Sn1—O1	140.42 (12)
C18—Sn1—O1	89.30 (18)
N1—Sn1—O1	69.60 (12)
C11—Sn1—O4	84.9 (2)
O3—Sn1—O4	78.79 (13)
C18—Sn1—O4	86.28 (19)
N1—Sn1—O4	149.60 (14)
O1—Sn1—O4	140.79 (12)
C11—Sn1—O1^i^	80.32 (18)
O3—Sn1—O1^i^	154.13 (12)
C18—Sn1—O1^i^	83.72 (16)
N1—Sn1—O1^i^	135.04 (12)
O1—Sn1—O1^i^	65.45 (12)
O4—Sn1—O1^i^	75.34 (11)

Symmetry code: (i) 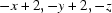.

**Table 2:** Hydrogen-bond geometry (Å, °)

*D*—H⋯*A*	*D*—H	H⋯*A*	*D*⋯*A*	*D*—H⋯*A*
O4—H4⋯O2^i^	0.82	1.82	2.624 (6)	165

Symmetry code: (i) 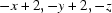.

**Fig. 1.**
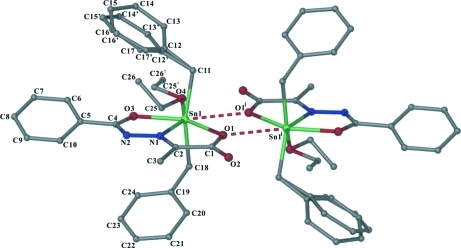
The molecular structure of the compound, showing 50% probability displacement ellipsoids. H atoms have been omitted for clarity. Symmetry code: 2 - x, 2 - y, -z.

**Fig. 2.**
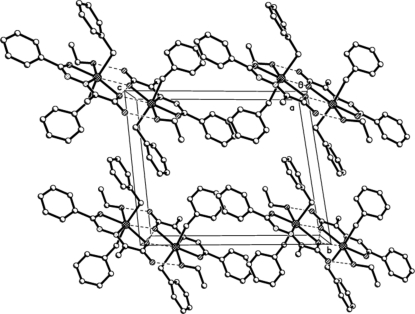
The crystal packing in a unit cell of the title complex, viewed along the b axis. H atoms have been omitted.

## supplementary crystallographic information

### Comment

Organotin derivatives of carboxylic acid ligands have been extensively studied due to their biological activities (Gielen *et al.*, 2002). In our ongoing studies with Schiff base organotin(IV) compounds, the title compound has been synthesized and we report herein its crystal structure. The molecular structure of the compound is shown in Fig.1. The atoms O1, O3, N1 and O4 are coplanar within 0.0120 Å, which form the equatorial plane. Furthermore, the angle of the axial C11—Sn1—C18 is 163.3 (3)°, which deviates from the linear angle of 180°. These data indicate that the tin atom of this complex is in a distorted octahedral configuration. The O1 atom of the carboxylate residue also binds the other tin atom, Sn^i^, generating a Sn_2_O_2_ four-membered ring [symmetry code: 2 - *x*, 2 - *y*, -*z*]. The distances of Sn1–O1^i^ 2.772 (4)Å are relatively longer than those of Sn1—O1 2.339 (4)Å (Table 1), but are comparable with those found in related seven-coordinate diorganotin systems (Sun *et al.*, 2007). With weak interactions of Sn–O bonding, the structure of the title complex can be described as a dimer with crystallographically imposed 1 symmetry. and the coordination geometry of tin can be also described as a *trans*-C_2_SnO_4_N pentagonal bipyramid with the two benzyl groups occupying *trans* positions. The forming of the dimer leads to the shorter interaction between O and O^i^, because the interaction of two monomers surpass the repelling effect of two O atoms. Otherwise, there exhibit the disorder at the C12 to C17 aromatic ring moiety and the C25, C26 atoms of the coordinated ethonal solvate molecule.

Each Sn atom is also coordinated by an ethanol molecule, the Sn1—O4 bond distance being 2.424 (3) Å, which is comparable with those in the analogous (Sun *et al.*, 2007), due to the formation of intradimeric hydrogen bonds, O2–O4^i^ (or O2^i^–O4) 2.624 (6)Å (Table 2). These hydrogen bonds contribute to the stability and compactness of the crystal structure (Fig. 2).

### Experimental

Pyruvic acid benzoyldrazone (1 mmol) and sodium ethoxide (1 mmol) was added to the solution of dry benzene (20 ml) in a Schlenk flask and stirred for 0.5 h. Dibenzyltin dichloride (1 mmol) was then added and the reaction mixture was stirred for 12 h at 313 K and then filtered. The solvent was gradually removed by evaporation under vacuum until a solid product was obtained. The solid was then recrystallized from ethanol and colorless crystals suitable for X-ray diffraction were obtained. Elemental analysis, calculated for C_26_H_28_N_2_O_4_Sn: C 56.66, H 5.12, N 5.08; found: C 56.51, H 5.34, N 5.01%.

### Refinement

The atoms C12, C13, C14, C15, C16 and C17 of the phenyl ring, C25 and C26 of the ethanol molecule were found to be disordered over two sites, and the ratio of the occupancy factors were refined to 0.57 (5):0.43 (5) and 0.79 (2):0.21 (2) for the phenyl ring C atoms and ethanol C atoms, respectively. The H atoms were positioned geometrically with aromatic C—H distances of 0.93 Å, and refined as riding on their parent atoms, with *U*_iso_(H) = 1.2 *U*_eq_(C, O). All other H atoms were also placed in idealized positions, with *U*_iso_(H) = 1.5 *U*_eq_(C, O).

### Crystal data

**Table:**

[Sn_2_(C_7_H_7_)_4_(C_10_H_8_N_2_O_3_)_2_(C_2_H_6_O)_2_]	*Z* = 1
*M**_r_* = 1102.42	*F*(000) = 560
Triclinic, *P*1	*D*_x_ = 1.468 Mg m^−^^3^
*a* = 8.7187 (18) Å	Mo *K*α radiation, λ = 0.71073 Å
*b* = 11.385 (2) Å	Cell parameters from 3583 reflections
*c* = 13.198 (3) Å	θ = 2.6–27.3°
α = 96.170 (3)°	µ = 1.06 mm^−^^1^
β = 93.728 (2)°	*T* = 298 K
γ = 105.861 (3)°	Block, colorless
*V* = 1246.8 (4) Å^3^	0.45 × 0.37 × 0.23 mm

### Data collection

**Table:**

Siemens SMART CCD area-detector diffractometer	4356 independent reflections
Radiation source: fine-focus sealed tube	3598 reflections with *I* > 2σ(*I*)
graphite	*R*_int_ = 0.017
phi and ω scans	θ_max_ = 25.0°, θ_min_ = 2.3°
Absorption correction: multi-scan (*SADABS*; Sheldrick, 1996)	*h* = −10→10
*T*_min_ = 0.647, *T*_max_ = 0.793	*k* = −13→12
6566 measured reflections	*l* = −15→15

### Refinement

**Table:**

Refinement on *F*^2^	Primary atom site location: structure-invariant direct methods
Least-squares matrix: full	Secondary atom site location: difference Fourier map
*R*[*F*^2^ > 2σ(*F*^2^)] = 0.042	Hydrogen site location: inferred from neighbouring sites
*wR*(*F*^2^) = 0.116	H-atom parameters constrained
*S* = 1.08	*w* = 1/[σ^2^(*F*_o_^2^) + (0.0509*P*)^2^ + 2.1936*P*] where *P* = (*F*_o_^2^ + 2*F*_c_^2^)/3
4356 reflections	(Δ/σ)_max_ = 0.001
298 parameters	Δρ_max_ = 1.04 e Å^−^^3^
0 restraints	Δρ_min_ = −0.58 e Å^−^^3^

### Fractional atomic coordinates and isotropic or equivalent isotropic displacement parameters (Å^2^)

**Table:**

	*x*	*y*	*z*	*U*_iso_*/*U*_eq_	Occ. (<1)
Sn1	0.93329 (4)	0.91659 (3)	0.13554 (2)	0.04906 (15)	
N1	0.7188 (5)	0.9747 (4)	0.1792 (3)	0.0470 (9)	
N2	0.6493 (5)	0.9324 (4)	0.2637 (3)	0.0512 (10)	
O1	0.8691 (4)	1.0434 (3)	0.0211 (2)	0.0504 (8)	
O2	0.7086 (5)	1.1605 (4)	−0.0098 (3)	0.0724 (12)	
O3	0.8542 (4)	0.8390 (3)	0.2709 (2)	0.0538 (9)	
O4	1.1210 (5)	0.8036 (4)	0.1661 (3)	0.0688 (11)	
H4	1.1866	0.8112	0.1237	0.103*	
C1	0.7536 (6)	1.0884 (5)	0.0400 (4)	0.0509 (12)	
C2	0.6661 (6)	1.0494 (5)	0.1312 (4)	0.0506 (12)	
C3	0.5333 (7)	1.0990 (6)	0.1618 (4)	0.0679 (16)	
H3A	0.4925	1.0645	0.2212	0.102*	
H3B	0.4492	1.0778	0.1066	0.102*	
H3C	0.5720	1.1870	0.1774	0.102*	
C4	0.7284 (6)	0.8633 (5)	0.3039 (4)	0.0537 (12)	
C5	0.6677 (6)	0.8078 (5)	0.3953 (4)	0.0569 (13)	
C6	0.5814 (8)	0.8628 (6)	0.4586 (4)	0.0730 (17)	
H6	0.5608	0.9355	0.4441	0.088*	
C7	0.5253 (9)	0.8109 (7)	0.5433 (5)	0.0824 (19)	
H7	0.4656	0.8484	0.5851	0.099*	
C8	0.5554 (9)	0.7072 (7)	0.5662 (5)	0.086 (2)	
H8	0.5173	0.6736	0.6240	0.104*	
C9	0.6420 (8)	0.6504 (7)	0.5052 (5)	0.0817 (19)	
H9	0.6628	0.5783	0.5210	0.098*	
C10	0.6986 (8)	0.7022 (6)	0.4190 (4)	0.0717 (16)	
H10	0.7580	0.6644	0.3772	0.086*	
C11	0.8119 (8)	0.7607 (5)	0.0268 (4)	0.0695 (16)	
H11A	0.7330	0.7837	−0.0161	0.083*	
H11B	0.8892	0.7414	−0.0170	0.083*	
C12	0.73 (9)	0.65 (8)	0.07 (6)	0.08 (4)	0.57 (5)
C13	0.804 (3)	0.5516 (19)	0.065 (3)	0.084 (6)	0.57 (5)
H13	0.8992	0.5572	0.0352	0.100*	0.57 (5)
C14	0.723 (3)	0.4437 (19)	0.110 (2)	0.087 (6)	0.57 (5)
H14	0.7650	0.3766	0.1083	0.105*	0.57 (5)
C15	0.582 (4)	0.440 (4)	0.156 (2)	0.090 (9)	0.57 (5)
H15	0.5369	0.3732	0.1897	0.108*	0.57 (5)
C16	0.504 (4)	0.533 (3)	0.1544 (19)	0.087 (8)	0.57 (5)
H16	0.4062	0.5260	0.1805	0.105*	0.57 (5)
C17	0.584 (7)	0.638 (5)	0.110 (3)	0.080 (8)	0.57 (5)
H17	0.5378	0.7020	0.1088	0.096*	0.57 (5)
C12'	0.71 (12)	0.66 (11)	0.08 (7)	0.08 (5)	0.43 (5)
C13'	0.771 (4)	0.562 (3)	0.116 (3)	0.085 (8)	0.43 (5)
H13'	0.8775	0.5638	0.1114	0.101*	0.43 (5)
C14'	0.668 (5)	0.466 (4)	0.159 (4)	0.087 (12)	0.43 (5)
H14'	0.7032	0.4010	0.1792	0.105*	0.43 (5)
C15'	0.510 (7)	0.472 (3)	0.172 (3)	0.085 (10)	0.43 (5)
H15'	0.4456	0.4140	0.2079	0.102*	0.43 (5)
C16'	0.447 (5)	0.562 (4)	0.133 (3)	0.087 (9)	0.43 (5)
H16'	0.3407	0.5608	0.1378	0.105*	0.43 (5)
C17'	0.551 (9)	0.654 (6)	0.086 (4)	0.080 (10)	0.43 (5)
H17'	0.5130	0.7144	0.0590	0.096*	0.43 (5)
C18	1.1115 (7)	1.0740 (5)	0.2175 (4)	0.0601 (14)	
H18A	1.1875	1.0456	0.2581	0.072*	
H18B	1.1696	1.1219	0.1684	0.072*	
C19	1.0435 (7)	1.1550 (6)	0.2859 (4)	0.0598 (14)	
C20	1.0187 (8)	1.2612 (6)	0.2555 (5)	0.0770 (17)	
H20	1.0463	1.2836	0.1919	0.092*	
C21	0.9518 (9)	1.3352 (7)	0.3206 (6)	0.092 (2)	
H21	0.9346	1.4069	0.3007	0.110*	
C22	0.9123 (9)	1.3008 (8)	0.4137 (6)	0.094 (2)	
H22	0.8676	1.3497	0.4569	0.113*	
C23	0.9365 (9)	1.1978 (8)	0.4443 (5)	0.088 (2)	
H23	0.9088	1.1763	0.5081	0.105*	
C24	1.0019 (8)	1.1246 (6)	0.3818 (4)	0.0721 (16)	
H24	1.0187	1.0537	0.4036	0.086*	
C25	1.2109 (15)	0.7949 (13)	0.2607 (7)	0.075 (3)	0.79 (2)
H25A	1.1945	0.8530	0.3153	0.090*	0.79 (2)
H25B	1.3244	0.8163	0.2518	0.090*	0.79 (2)
C26	1.1585 (19)	0.6675 (12)	0.2895 (11)	0.110 (5)	0.79 (2)
H26A	1.0507	0.6509	0.3080	0.165*	0.79 (2)
H26B	1.2283	0.6609	0.3466	0.165*	0.79 (2)
H26C	1.1629	0.6091	0.2324	0.165*	0.79 (2)
C25'	1.120 (7)	0.719 (5)	0.238 (3)	0.082 (12)	0.21 (2)
H25C	1.0292	0.6468	0.2190	0.099*	0.21 (2)
H25D	1.1083	0.7569	0.3053	0.099*	0.21 (2)
C26'	1.274 (7)	0.681 (4)	0.241 (4)	0.110 (18)	0.21 (2)
H26D	1.2485	0.5930	0.2267	0.165*	0.21 (2)
H26E	1.3307	0.7075	0.3082	0.165*	0.21 (2)
H26F	1.3391	0.7184	0.1911	0.165*	0.21 (2)

### Atomic displacement parameters (Å^2^)

**Table:**

	*U*^11^	*U*^22^	*U*^33^	*U*^12^	*U*^13^	*U*^23^
Sn1	0.0480 (2)	0.0633 (3)	0.0411 (2)	0.02061 (17)	0.01282 (14)	0.01142 (15)
N1	0.040 (2)	0.063 (3)	0.039 (2)	0.0126 (19)	0.0118 (17)	0.0094 (19)
N2	0.042 (2)	0.069 (3)	0.042 (2)	0.012 (2)	0.0105 (18)	0.010 (2)
O1	0.0480 (19)	0.069 (2)	0.0437 (18)	0.0262 (17)	0.0145 (15)	0.0146 (16)
O2	0.086 (3)	0.099 (3)	0.061 (2)	0.058 (3)	0.033 (2)	0.037 (2)
O3	0.052 (2)	0.068 (2)	0.0473 (19)	0.0206 (18)	0.0159 (16)	0.0149 (17)
O4	0.070 (3)	0.096 (3)	0.059 (2)	0.042 (2)	0.0245 (19)	0.030 (2)
C1	0.050 (3)	0.063 (3)	0.045 (3)	0.025 (3)	0.008 (2)	0.008 (2)
C2	0.047 (3)	0.065 (3)	0.044 (3)	0.020 (2)	0.011 (2)	0.010 (2)
C3	0.062 (4)	0.093 (4)	0.062 (3)	0.037 (3)	0.023 (3)	0.018 (3)
C4	0.057 (3)	0.064 (3)	0.041 (3)	0.016 (3)	0.011 (2)	0.011 (2)
C5	0.051 (3)	0.075 (4)	0.044 (3)	0.011 (3)	0.012 (2)	0.016 (3)
C6	0.076 (4)	0.094 (5)	0.052 (3)	0.021 (4)	0.023 (3)	0.020 (3)
C7	0.083 (5)	0.105 (5)	0.058 (4)	0.017 (4)	0.027 (3)	0.019 (4)
C8	0.085 (5)	0.106 (6)	0.061 (4)	0.003 (4)	0.021 (3)	0.029 (4)
C9	0.083 (5)	0.085 (5)	0.073 (4)	0.007 (4)	0.017 (4)	0.030 (4)
C10	0.073 (4)	0.084 (4)	0.058 (3)	0.017 (3)	0.015 (3)	0.018 (3)
C11	0.082 (4)	0.070 (4)	0.054 (3)	0.018 (3)	0.009 (3)	0.004 (3)
C12	0.09 (11)	0.07 (8)	0.06 (9)	0.02 (6)	0.00 (5)	0.01 (6)
C13	0.099 (12)	0.076 (10)	0.074 (14)	0.024 (8)	−0.004 (10)	0.010 (10)
C14	0.103 (13)	0.078 (11)	0.075 (14)	0.018 (9)	−0.004 (10)	0.011 (9)
C15	0.10 (3)	0.083 (19)	0.077 (12)	0.013 (17)	−0.002 (17)	0.016 (12)
C16	0.103 (19)	0.079 (19)	0.073 (14)	0.015 (16)	−0.002 (11)	0.015 (12)
C17	0.09 (2)	0.075 (16)	0.066 (19)	0.014 (13)	0.001 (14)	0.011 (12)
C12'	0.09 (16)	0.07 (10)	0.06 (12)	0.02 (8)	0.00 (8)	0.01 (8)
C13'	0.098 (16)	0.079 (14)	0.072 (17)	0.018 (11)	−0.004 (13)	0.015 (14)
C14'	0.10 (3)	0.08 (2)	0.074 (19)	0.011 (18)	0.00 (2)	0.018 (15)
C15'	0.10 (3)	0.08 (2)	0.074 (15)	0.020 (19)	−0.003 (15)	0.017 (15)
C16'	0.10 (2)	0.081 (18)	0.073 (16)	0.014 (14)	−0.001 (14)	0.012 (12)
C17'	0.09 (3)	0.075 (19)	0.07 (3)	0.014 (15)	0.001 (19)	0.011 (15)
C18	0.053 (3)	0.076 (4)	0.053 (3)	0.020 (3)	0.008 (2)	0.009 (3)
C19	0.052 (3)	0.072 (4)	0.051 (3)	0.013 (3)	0.004 (2)	0.004 (3)
C20	0.075 (4)	0.084 (5)	0.070 (4)	0.023 (4)	0.004 (3)	0.000 (3)
C21	0.089 (5)	0.090 (5)	0.093 (5)	0.027 (4)	−0.001 (4)	−0.004 (4)
C22	0.084 (5)	0.104 (6)	0.087 (5)	0.023 (4)	0.015 (4)	−0.021 (5)
C23	0.080 (5)	0.100 (6)	0.070 (4)	0.010 (4)	0.017 (4)	−0.009 (4)
C24	0.069 (4)	0.082 (4)	0.058 (3)	0.012 (3)	0.011 (3)	0.001 (3)
C25	0.073 (7)	0.090 (8)	0.069 (6)	0.026 (6)	0.015 (5)	0.025 (5)
C26	0.127 (11)	0.117 (9)	0.099 (9)	0.049 (8)	0.005 (8)	0.033 (7)
C25'	0.09 (3)	0.08 (3)	0.07 (2)	0.03 (3)	0.00 (2)	0.02 (2)
C26'	0.13 (4)	0.12 (3)	0.10 (3)	0.05 (3)	0.01 (3)	0.03 (2)

### Geometric parameters (Å, °)

**Table:**

Sn1—C11	2.135 (6)	C15—C16	1.40 (4)
Sn1—O3	2.148 (3)	C15—H15	0.9300
Sn1—C18	2.154 (6)	C16—C17	1.41 (6)
Sn1—N1	2.237 (4)	C16—H16	0.9300
Sn1—O1	2.341 (3)	C17—H17	0.9300
Sn1—O4	2.382 (4)	C12'—C13'	1.4 (12)
Sn1—O1^i^	2.772 (3)	C12'—C17'	1.4 (9)
N1—C2	1.276 (6)	C13'—C14'	1.41 (4)
N1—N2	1.373 (5)	C13'—H13'	0.9300
N2—C4	1.310 (7)	C14'—C15'	1.41 (6)
O1—C1	1.276 (6)	C14'—H14'	0.9300
O2—C1	1.233 (6)	C15'—C16'	1.42 (4)
O3—C4	1.293 (6)	C15'—H15'	0.9300
O4—C25'	1.42 (4)	C16'—C17'	1.42 (9)
O4—C25	1.458 (10)	C16'—H16'	0.9300
O4—H4	0.8200	C17'—H17'	0.9300
C1—C2	1.508 (7)	C18—C19	1.485 (8)
C2—C3	1.481 (7)	C18—H18A	0.9700
C3—H3A	0.9600	C18—H18B	0.9700
C3—H3B	0.9600	C19—C20	1.380 (9)
C3—H3C	0.9600	C19—C24	1.392 (8)
C4—C5	1.483 (7)	C20—C21	1.403 (9)
C5—C10	1.364 (8)	C20—H20	0.9300
C5—C6	1.372 (8)	C21—C22	1.366 (10)
C6—C7	1.376 (8)	C21—H21	0.9300
C6—H6	0.9300	C22—C23	1.345 (11)
C7—C8	1.338 (10)	C22—H22	0.9300
C7—H7	0.9300	C23—C24	1.369 (9)
C8—C9	1.368 (10)	C23—H23	0.9300
C8—H8	0.9300	C24—H24	0.9300
C9—C10	1.394 (8)	C25—C26	1.49 (2)
C9—H9	0.9300	C25—H25A	0.9700
C10—H10	0.9300	C25—H25B	0.9700
C11—C12	1.5 (9)	C26—H26A	0.9600
C11—C12'	1.5 (12)	C26—H26B	0.9600
C11—H11A	0.9700	C26—H26C	0.9600
C11—H11B	0.9700	C25'—C26'	1.52 (7)
C12—C17	1.4 (6)	C25'—H25C	0.9700
C12—C13	1.4 (7)	C25'—H25D	0.9700
C13—C14	1.45 (3)	C26'—H26D	0.9600
C13—H13	0.9300	C26'—H26E	0.9600
C14—C15	1.41 (3)	C26'—H26F	0.9600
C14—H14	0.9300		
			
C11—Sn1—O3	97.42 (19)	C17—C12—C11	121 (10)
C11—Sn1—C18	163.3 (2)	C13—C12—C11	117 (10)
O3—Sn1—C18	94.77 (18)	C12—C13—C14	116 (10)
C11—Sn1—N1	97.9 (2)	C12—C13—H13	122.0
O3—Sn1—N1	70.83 (13)	C14—C13—H13	122.0
C18—Sn1—N1	96.85 (18)	C15—C14—C13	120 (2)
C11—Sn1—O1	88.59 (19)	C15—C14—H14	120.0
O3—Sn1—O1	140.42 (12)	C13—C14—H14	120.0
C18—Sn1—O1	89.30 (18)	C16—C15—C14	123 (3)
N1—Sn1—O1	69.60 (12)	C16—C15—H15	118.3
C11—Sn1—O4	84.9 (2)	C14—C15—H15	118.3
O3—Sn1—O4	78.79 (13)	C15—C16—C17	116 (3)
C18—Sn1—O4	86.28 (19)	C15—C16—H16	122.0
N1—Sn1—O4	149.60 (14)	C17—C16—H16	122.0
O1—Sn1—O4	140.79 (12)	C12—C17—C16	122 (10)
C11—Sn1—O1^i^	80.32 (18)	C12—C17—H17	118.8
O3—Sn1—O1^i^	154.13 (12)	C16—C17—H17	118.8
C18—Sn1—O1^i^	83.72 (16)	C13'—C12'—C17'	120 (10)
N1—Sn1—O1^i^	135.04 (12)	C13'—C12'—C11	122.0
O1—Sn1—O1^i^	65.45 (12)	C17'—C12'—C11	119.0
O4—Sn1—O1^i^	75.34 (11)	C12'—C13'—C14'	121 (10)
C2—N1—N2	120.3 (4)	C12'—C13'—H13'	119.7
C2—N1—Sn1	121.9 (3)	C14'—C13'—H13'	119.7
N2—N1—Sn1	117.7 (3)	C13'—C14'—C15'	118 (3)
C4—N2—N1	109.7 (4)	C13'—C14'—H14'	120.9
C1—O1—Sn1	117.0 (3)	C15'—C14'—H14'	120.9
C4—O3—Sn1	115.9 (3)	C14'—C15'—C16'	122 (4)
C25'—O4—C25	41 (2)	C14'—C15'—H15'	118.9
C25'—O4—Sn1	128.5 (18)	C16'—C15'—H15'	118.9
C25—O4—Sn1	130.5 (5)	C17'—C16'—C15'	118 (4)
C25'—O4—H4	119.4	C17'—C16'—H16'	121.1
C25—O4—H4	104.5	C15'—C16'—H16'	121.1
Sn1—O4—H4	111.7	C16'—C17'—C12'	121 (10)
O2—C1—O1	125.3 (5)	C16'—C17'—H17'	119.7
O2—C1—C2	118.0 (4)	C12'—C17'—H17'	119.7
O1—C1—C2	116.7 (4)	C19—C18—Sn1	113.5 (4)
N1—C2—C3	124.2 (5)	C19—C18—H18A	108.9
N1—C2—C1	114.7 (4)	Sn1—C18—H18A	108.9
C3—C2—C1	121.1 (5)	C19—C18—H18B	108.9
C2—C3—H3A	109.5	Sn1—C18—H18B	108.9
C2—C3—H3B	109.5	H18A—C18—H18B	107.7
H3A—C3—H3B	109.5	C20—C19—C24	118.6 (6)
C2—C3—H3C	109.5	C20—C19—C18	120.8 (5)
H3A—C3—H3C	109.5	C24—C19—C18	120.6 (6)
H3B—C3—H3C	109.5	C19—C20—C21	119.8 (7)
O3—C4—N2	125.9 (4)	C19—C20—H20	120.1
O3—C4—C5	117.2 (5)	C21—C20—H20	120.1
N2—C4—C5	116.9 (5)	C22—C21—C20	119.2 (8)
C10—C5—C6	118.8 (5)	C22—C21—H21	120.4
C10—C5—C4	120.7 (5)	C20—C21—H21	120.4
C6—C5—C4	120.5 (5)	C23—C22—C21	121.5 (7)
C5—C6—C7	120.3 (7)	C23—C22—H22	119.3
C5—C6—H6	119.8	C21—C22—H22	119.3
C7—C6—H6	119.8	C22—C23—C24	120.1 (7)
C8—C7—C6	120.8 (7)	C22—C23—H23	119.9
C8—C7—H7	119.6	C24—C23—H23	119.9
C6—C7—H7	119.6	C23—C24—C19	120.8 (7)
C7—C8—C9	120.4 (6)	C23—C24—H24	119.6
C7—C8—H8	119.8	C19—C24—H24	119.6
C9—C8—H8	119.8	O4—C25—C26	110.9 (11)
C8—C9—C10	119.1 (7)	O4—C25—H25A	109.5
C8—C9—H9	120.5	C26—C25—H25A	109.5
C10—C9—H9	120.5	O4—C25—H25B	109.5
C5—C10—C9	120.6 (6)	C26—C25—H25B	109.5
C5—C10—H10	119.7	H25A—C25—H25B	108.1
C9—C10—H10	119.7	O4—C25'—C26'	111 (4)
C12—C11—C12'	8(10)	O4—C25'—H25C	109.5
C12—C11—Sn1	116 (10)	C26'—C25'—H25C	109.5
C12'—C11—Sn1	112 (10)	O4—C25'—H25D	109.5
C12—C11—H11A	108.3	C26'—C25'—H25D	109.5
C12'—C11—H11A	104.2	H25C—C25'—H25D	108.1
Sn1—C11—H11A	108.2	C25'—C26'—H26D	109.5
C12—C11—H11B	108.2	C25'—C26'—H26E	109.5
C12'—C11—H11B	119.0	H26D—C26'—H26E	109.5
Sn1—C11—H11B	108.2	C25'—C26'—H26F	109.5
H11A—C11—H11B	107.4	H26D—C26'—H26F	109.5
C17—C12—C13	122 (10)	H26E—C26'—H26F	109.5

Symmetry codes: (i) −*x*+2, −*y*+2, −*z*.

### Hydrogen-bond geometry (Å, °)

**Table:**

*D*—H···*A*	*D*—H	H···*A*	*D*···*A*	*D*—H···*A*
O4—H4···O2^i^	0.82	1.82	2.624 (6)	165

Symmetry codes: (i) −*x*+2, −*y*+2, −*z*.

## References

[bb1] Gielen, M., Vanbellinghen, C., Gelan, J. & Willem, R. (2002). *Bull. Soc. Chim. Belg.* **97**, 873–878.

[bb2] Sheldrick, G. M. (1996). *SADABS* University of Göttingen, Germany.

[bb3] Sheldrick, G. M. (2008). *Acta Cryst.* A**64**, 112–122. 10.1107/S0108767307043930 18156677

[bb4] Siemens (1996). *SMART* and *SAINT* Siemens Analytical X-ray Instruments Inc., Madison, Wisconsin, USA.

[bb5] Sun, L.-N. & Hu, C.-W. (2007). *Acta Cryst.* E**63**, m1832–m1833.

